# Supplementary figures and images for: Great-tailed Grackles (Quiscalus mexicanus) as a tolerant host of avian malaria parasites
Source: PLoS One. 2022 Aug 23;17(8):e0268161. doi: 10.1371/journal.pone.0268161 (PMC9397854; doi:10.1371/journal.pone.0268161)

A

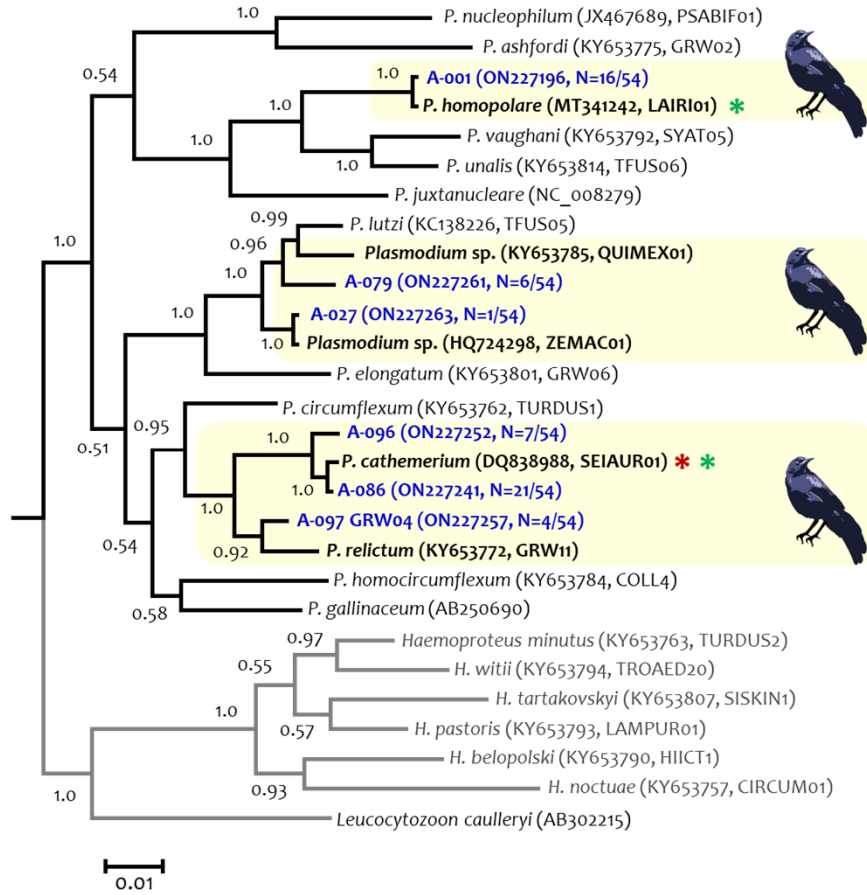

B

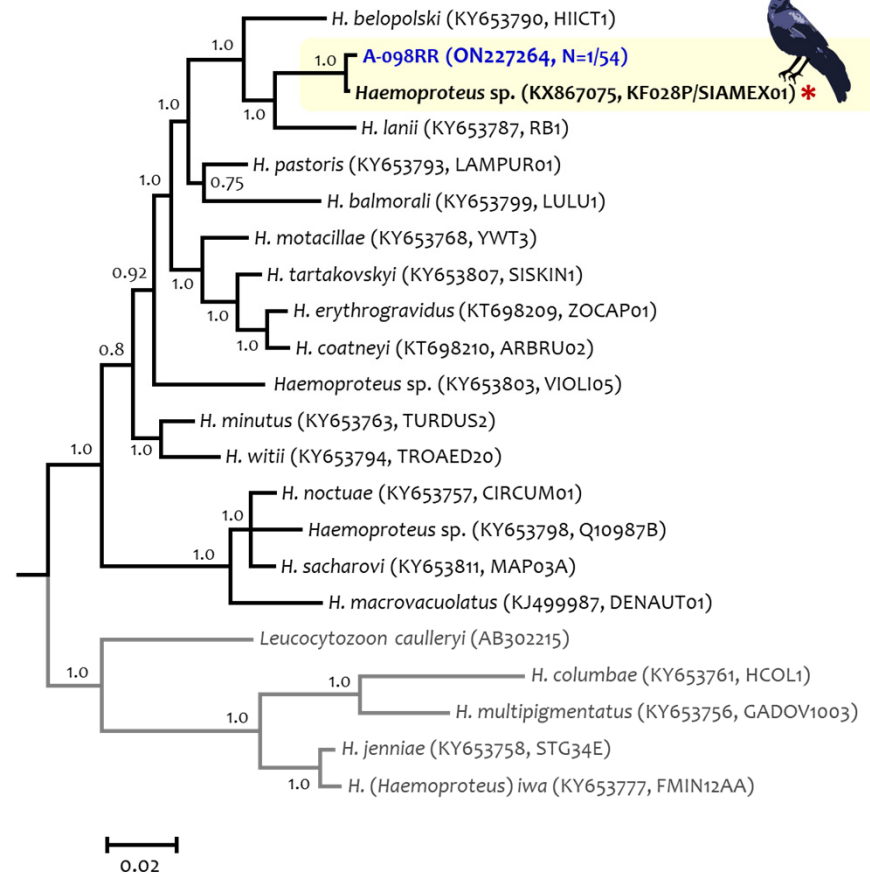

Supplement: S1 Fig — Phylogenetic trees were computed based on larger fragments of (A) Plasmodium sequences of cytb gene (945 bp out of the 1,134 bp of cytb gene, excluding gaps) and (B) Haemoproteus sequences of cytb gene (1,014 bp out of the 1,134 bp of cytb gene, excluding gaps). Values above branches are posterior probabilities. Leucocytozoon and Haemoproteus (Haemoproteus) genera (outgroup) are indicated in grey. Genbank accession numbers and lineage identifiers, as deposited in the MalAvi database, are provided in parenthesis for the sequences used in the analyses. Plasmodium and Haemoproteus (Parahaemoproteus) recovered from grackles from Arizona are written in blue. Lineages detected in grackles from Texas [37] are indicated with a red asterisk and from Mexico with a green asterisk. (PDF) [file pone.0268161.s001.pdf]
