# Supplementary material for: Great-tailed Grackles (Quiscalus mexicanus) as a tolerant host of avian malaria parasites
Source: PLoS One. 2022 Aug 23;17(8):e0268161. doi: 10.1371/journal.pone.0268161 (PMC9397854; doi:10.1371/journal.pone.0268161)
Supplement: S2 Table — (PDF) [file pone.0268161.s003.pdf]

Table S2A. Estimates of evolutionary divergence between *cytb Plasmodium* sequences. Standard error estimates are shown above the diagonal. The analysis involved 23 nucleotide sequences and all codon positions There were a total of 454 positions in the final dataset. Evolutionary analyses were conducted in MEGA7 [1]. GenBank accession numbers and linages name are shown in parenthesis.

		Genetic distance (Standard error estimates)																								
	**Parasite lineages**	**1**	**2**	**3**	**4**	**5**	**6**	**7**	**8**	**9**	**10**	**11**	**12**	**13**	**14**	**15**	**16**	**17**	**18**	**19**	**20**	**21**	**22**	**23**	**24**	**25**
**1**	*P. homopolare* (KJ482708, BAEBIC02)		**0.004**	**0.004**	0.009	0.011	0.011	0.012	0.012	0.012	0.012	0.012	0.012	0.012	0.011	0.012	0.012	0.011	0.011	0.012	0.012	0.011	0.009	0.009	0.012	0.012
**2**	***P. homopolare*** (MT341242, LAIRI01)	**0.007**		**0.000**	0.009	0.011	0.011	0.011	0.011	0.011	0.011	0.012	0.012	0.012	0.011	0.012	0.012	0.011	0.011	0.011	0.011	0.011	0.008	0.009	0.012	0.011
**3**	**A-001 (ON227196, LAIRI01)**	**0.007**	**0.000**		0.009	0.011	0.011	0.011	0.011	0.011	0.011	0.012	0.012	0.012	0.011	0.012	0.012	0.011	0.011	0.011	0.011	0.011	0.008	0.009	0.012	0.011
**4**	*P. parahexamerium* (FJ389155, P-ALDI1)	0.037	0.040	0.040		0.012	0.012	0.012	0.012	0.012	0.012	0.012	0.012	0.012	0.011	0.012	0.012	0.011	0.011	0.011	0.012	0.011	0.010	0.009	0.013	0.012
**5**	***P. cathemerium*** (DQ838988, SEIAUR01)	0.066	0.064	0.064	0.075		**0.000**	**0.004**	**0.004**	0.008	0.008	0.008	0.011	0.008	0.008	0.011	0.011	0.009	0.009	0.010	0.009	0.010	0.011	0.011	0.011	0.012
**6**	**A-086 (ON227241, SEIAUR01)**	0.066	0.064	0.064	0.075	**0.000**		**0.004**	**0.004**	0.008	0.008	0.008	0.011	0.008	0.008	0.011	0.011	0.009	0.009	0.010	0.009	0.010	0.011	0.011	0.011	0.012
**7**	*Plasmodium* sp. (DQ659539, MOLATE01)	0.070	0.068	0.068	0.079	**0.009**	**0.009**		**0.000**	0.008	0.008	0.008	0.011	0.009	0.009	0.011	0.011	0.010	0.010	0.011	0.010	0.010	0.011	0.012	0.011	0.012
**8**	**A-096 (ON227252, MOLATE01)**	0.070	0.068	0.068	0.079	**0.009**	**0.009**	**0.000**		0.008	0.008	0.008	0.011	0.009	0.009	0.011	0.011	0.010	0.010	0.011	0.010	0.010	0.011	0.012	0.011	0.012
**9**	***P. relictum*** (AF254975, GRW04)	0.070	0.068	0.068	0.075	0.029	0.029	0.031	0.031		**0.000**	**0.006**	0.011	0.008	0.008	0.011	0.011	0.010	0.010	0.011	0.010	0.010	0.012	0.013	0.011	0.011
**10**	**A-097 (ON227257, GRW04)**	0.070	0.068	0.068	0.075	0.029	0.029	0.031	0.031	**0.000**		**0.006**	0.011	0.008	0.008	0.011	0.011	0.010	0.010	0.011	0.010	0.010	0.012	0.013	0.011	0.011
**11**	***P. relictum*** (KY653772, GRW11)	0.073	0.070	0.070	0.077	0.031	0.031	0.029	0.029	**0.020**	**0.020**		0.011	0.009	0.009	0.011	0.011	0.010	0.010	0.012	0.010	0.010	0.012	0.013	0.011	0.012
**12**	*P. lucens* (FJ389156, P-CYOL2)	0.077	0.075	0.075	0.075	0.053	0.053	0.062	0.062	0.051	0.051	0.055		0.010	0.011	0.012	0.012	0.012	0.012	0.012	0.011	0.012	0.012	0.012	0.012	0.012
**13**	*P. megaglobularis* (EU770152, P-CYOL1)	0.081	0.079	0.079	0.081	0.033	0.033	0.037	0.037	0.037	0.037	0.035	0.057		0.009	0.011	0.011	0.011	0.011	0.011	0.011	0.011	0.012	0.012	0.011	0.011
**14**	*P. circumfrexum* (KY653762, TURDUS1)	0.064	0.062	0.062	0.066	0.033	0.033	0.037	0.037	0.037	0.037	0.044	0.059	0.044		0.011	0.011	0.010	0.010	0.011	0.011	0.010	0.011	0.011	0.011	0.011
**15**	***Plasmodium*** sp. (EF153642, PHPAT01)	0.081	0.079	0.079	0.079	0.059	0.059	0.068	0.068	0.064	0.064	0.070	0.073	0.077	0.064		**0.000**	**0.006**	**0.006**	0.008	0.007	0.007	0.012	0.012	0.012	0.012
**16**	**A-079 (ON227261, PHPAT01)**	0.081	0.079	0.079	0.079	0.059	0.059	0.068	0.068	0.064	0.064	0.070	0.073	0.077	0.064	**0.000**		**0.006**	**0.006**	0.008	0.007	0.007	0.012	0.012	0.012	0.012
**17**	***Plasmodium*** sp. (HQ724298, ZEMAC01)	0.070	0.068	0.068	0.064	0.048	0.048	0.057	0.057	0.053	0.053	0.059	0.070	0.066	0.057	**0.015**	**0.015**		**0.000**	0.007	0.006	0.006	0.011	0.012	0.011	0.012
**18**	**A-027 (ON227263, ZEMAC01)**	0.070	0.068	0.068	0.064	0.048	0.048	0.057	0.057	0.053	0.053	0.059	0.070	0.066	0.057	**0.015**	**0.015**	**0.000**		0.007	0.006	0.006	0.011	0.012	0.011	0.012
**19**	*Plasmodium* sp. (KY653785, QUIMEX01)	0.077	0.075	0.075	0.073	0.055	0.055	0.064	0.064	0.066	0.066	0.068	0.077	0.073	0.070	0.029	0.029	0.026	0.026		0.007	0.007	0.012	0.011	0.012	0.013
**20**	*P. lutzi* (KC138226, TFUS05)	0.075	0.068	0.068	0.070	0.044	0.044	0.053	0.053	0.051	0.051	0.053	0.062	0.057	0.059	0.024	0.024	0.018	0.018	0.026		0.006	0.011	0.012	0.010	0.011
**21**	*P. matutinum* (KY287235, LINN1)	0.068	0.066	0.066	0.066	0.048	0.048	0.053	0.053	0.053	0.053	0.055	0.070	0.062	0.057	0.029	0.029	0.018	0.018	0.026	0.018		0.011	0.011	0.011	0.011
**22**	*P. unalis* (KY653814, TFUS06)	0.044	0.037	0.037	0.042	0.068	0.068	0.068	0.068	0.073	0.073	0.070	0.079	0.075	0.066	0.084	0.084	0.073	0.073	0.079	0.066	0.070		0.008	0.011	0.011
**23**	*P. vaughani* (KY653792, SYAT05)	0.048	0.046	0.046	0.042	0.068	0.068	0.077	0.077	0.077	0.077	0.079	0.075	0.084	0.064	0.088	0.088	0.077	0.077	0.075	0.075	0.075	0.033		0.013	0.011
**24**	*Plasmodium* sp. (ON262943, QUIMEX02)	0.073	0.070	0.070	0.079	0.064	0.064	0.068	0.068	0.070	0.070	0.068	0.081	0.064	0.059	0.075	0.075	0.064	0.064	0.077	0.057	0.062	0.070	0.081		0.008
**25**	*P. nucleophilum* (ON262942, DENPET03)	0.075	0.068	0.068	0.077	0.070	0.070	0.075	0.075	0.073	0.073	0.075	0.079	0.068	0.062	0.077	0.077	0.066	0.066	0.081	0.064	0.064	0.064	0.070	0.033	

Table S2B. Estimates of evolutionary divergence between *cytb Haemoproteus* sequences. Standard error estimates are shown above the diagonal. The analysis involved 5 nucleotide sequences and all codon positions. There were a total of 467 positions in the final dataset. Evolutionary analyses were conducted in MEGA7 [1]. GenBank accession numbers and linages name are shown in parenthesis.

		Genetic distance (Standard error estimates)				
	**Parasite lineages**	**1**	**2**	**3**	**4**	**5**
**1**	*Haemoproteus* sp. (AF465562, SIAMEX01)		**0.000**	**0.000**	0.003	0.008
**2**	**A-098 (ON227264, SIAMEX01)**	**0.000**		**0.000**	0.003	0.008
**3**	*Haemoproteus* sp. (KX867075, KF028P)	**0.000**	**0.000**		0.003	0.008
**4**	*Haemoproteus* sp. (MF077669, PIPMAC01)	0.004	0.004	0.004		0.009
**5**	*H. lanii* (KY653787_RB1)	0.039	0.039	0.039	0.039	

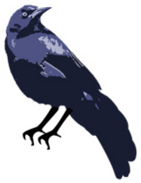

References:

1. Kumar S., Stecher G., and Tamura K. (2016). MEGA7: Molecular Evolutionary Genetics Analysis version 7.0 for bigger datasets. Molecular Biology and Evolution 33:1870-1874.
